# Regional heterogeneity in response of airway epithelial cells to cigarette smoke

**DOI:** 10.1186/s12890-018-0715-4

**Published:** 2018-09-04

**Authors:** Hario Baskoro, Tadashi Sato, Keiko Karasutani, Yohei Suzuki, Aki Mitsui, Naoko Arano, Fariz Nurwidya, Motoyasu Kato, Fumiyuki Takahashi, Yuzo Kodama, Kuniaki Seyama, Kazuhisa Takahashi

**Affiliations:** 1 Department of Respiratory Medicine, Juntendo University Graduate School of Medicine, 3-1-3 Hongo, Bunkyo-ku, Tokyo, 113-8431 Japan; 2 Department of Pulmonology and Respiratory Medicine, Universitas Indonesia Faculty of Medicine, Jalan Persahabatan Raya No. 1, Rawamangun, Jakarta, 13230 Indonesia

**Keywords:** Cigarette smoke, Airway epithelial cells, Small airway, Inflammation, Cyclooxygenase-2, Chronic obstructive pulmonary disease

## Abstract

**Background:**

Cigarette smoke (CS) exposure causes an abnormal inflammatory response, which can result in chronic obstructive pulmonary disease (COPD). Previous studies show that this disorder predominantly occurs in peripheral or small-airway areas, whereas the same condition has not been identified in the larger airways during the course of COPD. However, the different biochemical and genetic alterations occurring in response to CS exposure among airway epithelial cells from different sites in the lungs have not been fully investigated.

**Methods:**

Human small airway epithelial cells (SAECs) and normal human bronchial epithelial cells (NHBEs) were exposed to CS extract (CSE), and microarray analysis was used to determine gene- and protein-expression profiles and identify alterations following CSE exposure in both cell types. An in vivo smoking experiment was also performed to confirm differential responses to CS between sites in the lung.

**Results:**

Microarray analysis of SAECs and NHBEs following 24 h of CSE exposure showed that inflammatory related pathways and terms, including the tumor necrosis factor-signaling pathway, were overrepresented, especially in SAECs. Clustering analysis highlighted *prostaglandin-endoperoxide synthase-2* [also known as cyclooxygenase (COX)-2] as a gene specifically upregulated in SAECs, with COX-2 mRNA and protein expression significantly elevated by CSE exposure in SAECs (3.1- and 3.1-fold, respectively), but not in NHBEs. Furthermore, time-course analysis of COX-2 expression revealed earlier increases in SAECs compared with NHBEs following CS exposure. Short-term exposure of mouse lungs to CS was found to predominantly induce COX-2 expression in the small airway.

**Conclusions:**

The small airway is more susceptible to CSE than the large airway and could be the initial site of development of CS-related respiratory diseases, such as COPD.

**Electronic supplementary material:**

The online version of this article (10.1186/s12890-018-0715-4) contains supplementary material, which is available to authorized users.

## Background

Cigarette smoke (CS) produces oxidant and reactive oxygen species in the lungs [1] and the unfavorable impacts of CS represent a primary cause of respiratory diseases, such as chronic obstructive pulmonary disease (COPD) and a leading contributor to preventable morbidity and mortality [2]. Preventing CS-related diseases can be best achieved by avoiding smoking initiation [3]; however, when the smoking habit has already started, smoking cessation considerably decreases numerous adverse health effects [4]. Intervention by smoking cessation during the early stages of COPD can decrease the rate of lung-function loss [5]. Moreover, CS-induced oxidant burden can irreversibly change endogenous antioxidant protection in airways. CS-derived oxidants injure airway epithelial cells by affecting lipid-membrane, carbohydrate, protein, and DNA structures and lead to the persistent inflammation evident in COPD development [6].

In addition to its status as a leading cause of mortality worldwide, COPD is also the only major cause of death that continues to increase annually [2]. Two main features of COPD include progressive limitation of expiratory airflow, which is not completely reversible, and an abnormal inflammatory response in the lungs [7]. Hogg et al. [8] suggested that the initial locus of inflammation in COPD is the small airways represented by membranous (diameter, < 2 mm), terminal and respiratory bronchioles. In smokers, the small airways show structural abnormalities, with or without COPD. The relationship between inflammatory mucus exudates from airway lumen and COPD severity was investigated by analyzing lung tissue from patients with all clinical stages of COPD [9], revealing that small-airway obstruction associated with COPD is related to thickening of the airway wall as a consequence of the remodelling process marked by mucociliary clearance-component malfunction and tissue repair related to innate-immune mechanisms, finally resulting in acculumation of lumen inflammatory exudates [9, 10]. A more recent study from the same group, based on multidetector computed tomography obtained from patients at various stages of COPD, showed that narrowing and loss of small conducting airways occurs before the emphysematous changes associated with COPD [11]. However, the genetic and biochemical alterations involved in responses to CS exposure by airway epithelial cells from different sites in the lungs have not been fully investigated.

In this study, we investigated the response to CS in two commercially available human airway epithelial cell lines [small airway epithelial cells (SAECs) and normal human bronchial epithelial cells (NHBEs)]. SAECs were isolated from the distal portion of a healthy lung in the 1-mm bronchiole area, and NHBEs were derived from the epithelial lining of airways above the bifurcation of the lung (according to documentation from Lonza, Basel, Switzerland). We explored the genetic differences between SAECs and NHBEs during the initial phase of CS-exposure using microarray analysis and performed in vivo smoking experiments using a mouse model to confirm that cells from different lung sites respond differently to CS exposure.

## Methods

### Preparation of CS extract

CS extract (CSE) was prepared using commercially marketed Peace unfiltered cigarettes (29 mg of tar and 2.5 mg of nicotine per cigarette; Japan Tobacco, Tokyo, Japan). The smoke from one cigarette was bubbled through 15 mL of sterilized purified water, and this solution passed through a 0.22-μm pore filter (Pall Corporation, Port Washington, NY, USA), to generate 100% CSE. Pilot cell-growth experiments showed that concentrations of ≤2.5% CSE were not cytotoxic; therefore, cultured cells were exposed to 2.5% CSE.

### Cell culture

SAECs and NHBEs were purchased from Lonza. Three batches of each cell type, each originally isolated from three different healthy never-smoker donors, were used. SAECs were cultured with small airway epithelial cell growth medium (Lonza), and NHBEs were cultured with bronchial epithelial cell growth medium (Lonza), according to the manufacturer’s instructions (SAECs and NHBEs were seeded at 2500 and 3500 cells/cm^2^, respectively, in 25 cm^2^ plastic flasks). Both cell types were used at passages three to six and assayed at the same passages for direct comparisons. After reaching approximately 80% confluence, cells were treated with or without 2.5% CSE until assayed.

### RNA extraction and real-time quantitative polymerase chain reaction (qPCR)

Total RNA was extracted using miRNeasy mini kits (Qiagen, Hilden, Germany). For cDNA synthesis, total RNA was transcribed using ReverTra Ace qPCR RT master mix with gDNA remover (Toyobo, Osaka, Japan). Real-time qPCR for *cyclooxygenase* (*COX*)*-2* mRNA detection was performed using Thunderbird SYBR qPCR mix (Toyobo), an ABI 7500 Fast real-time PCR instrument (Applied Biosystems, Foster City, CA, USA), and *COX-2*-specific primers [(5′ → 3′) Fw: CTGGCGCTCAGCCATACAG; Rv: CCGGGTACAATCGCACTTATACT; Thermo Fisher Scientific, Waltham, MA, USA] according to manufacturer instructions. The expression of β-actin was measured using specific primers [(5′ → 3′) Fw: CTCTTCCAGCCTTCCTTCCT; Rv: AGCACTGTGTTGGCGTACAG; Thermo Fisher Scientific] as an internal control.

### Microarray analysis

Two different batches of SAECs and NHBEs were used for microarray analysis. RNA samples (2 μg) were labeled using the low input Quick Amp labeling kit (Agilent Technologies, Waldbronn, Germany). Labeled RNA was hybridized to an oligonucleotide microarray (Whole Human Genome 4× 44 K; Agilent Technologies) at 60 °C for 17 h. After washing with reagents from the gene expression wash buffer kit (Agilent Technologies) and drying, slides were scanned with an Agilent microarray scanner and analyzed using Feature Extraction software (v9.5.1; Agilent Technologies). Normalization was performed using global normalization methods. Data from the microarray analysis have been deposited in the Gene Expression Omnibus database (accession ID: GSE107200). Differentially expressed genes were defined based on unpaired *t*-test *p* values < 0.01 and fold change ≥2. Gene-set enrichment analysis (GSEA) of differentially expressed genes was conducted using the DAVID functional annotation tool (https://david.ncifcrf.gov/home.jsp). Gene sets from the Kyoto Encyclopedia of Genes and Genomes (KEGG; http://www.genome.jp/kegg/) and the Gene Ontology (GO) biological process database (http://www.geneontology.org/) were used. Pathways and ontology terms with a *p* <  0.05 were considered significant. The Benjamini-Hochberg procedure was used in the analysis to control the false discovery rate.

### Western blot analysis

Whole-cell lysates were prepared, and the total protein content of each measured using a BCA kit (Thermo Fisher Scientific). Protein aliquots (10 μg) were separated by electrophoresis on 10% sodium dodecyl sulfate polyacrylamide gels. After transferring the proteins to Immobilion-P membranes (Merck Millipore Corporation, Darmstadt, Germany), membranes were blocked with 5% skim milk in Tris-buffered saline with Tween-20 and allowed to react with an anti-COX-2 (1:400; Santa Cruz Biotechnology, Dallas, TX, USA) and anti-β-actin (1:10,000; Wako, Tokyo, Japan) monoclonal antibodies at 4 °C overnight. Membranes were then incubated with the appropriate horseradish-peroxidase-conjugated secondary antibody (1:20,000; Sigma-Aldrich, St. Louis, MO, USA), followed by detection with the Clarity western enhanced chemiluminescence substrate (Bio-Rad Laboratories, Hercules, CA, USA). Western blot signals were captured using an ImageQuant LAS-4000 mini fluorescence imager (GE Healthcare, Pittsburgh, PA, USA) and quantified using Multi Gauge image analysis software (Fujifilm Corporation, Tokyo, Japan).

### In vivo exposure to CS and preparation of lung sections

C57BL/6 J male mice (3 months old; *n* = 6) were purchased from Oriental Yeast Co., Ltd. (Tokyo, Japan) and used for the smoking experiment. The procedures for animal experimentation were approved by the Animal Care and Use Committee of Juntendo University School of Medicine (Tokyo, Japan). Mice were maintained in a limited-access barrier facility and housed in a humidity (55% ± 10%) and temperature (24 °C ± 2 °C) controlled room under 12-h light/dark cycles. Mice were fed with standard commercial chow (CRF-1; Oriental Yeast) and provided water ad libitum. Mice were exposed to CS using Peace unfiltered cigarettes and a tobacco smoke-inhalation experimental system for small animals (Model SIS-CS; Shibata Scientific Technology, Tokyo, Japan) [12–15]. The experimental settings were as follows: 15 mL stroke volume, six puffs per minute, and 3.5% CS diluted with compressed air (the mass concentration of total particulate matter; 1430 ± 100 mg/m^3^). Mice were exposed to either diluted CS or fresh air (as a control, *n* = 3 each) for 30 min/day over 5 days.

Mice were sacrificed at day 5 and their lungs subsequently processed as described previously [13–15]. Briefly, mice were anesthetized and then sacrificed by exsanguination of the left atrium with perfusion of phosphate-buffered saline (PBS) through the main pulmonary artery. Lungs were inflated and fixed by intratracheal instillation of 10% buffered formalin (pH 7.4; Wako) at a constant pressure of 25 cm H_2_O for 48 h. Frontal plane sections of the lungs were taken at the depth of the hilum, followed by further sectioning of each block into two equal-sized pieces, and from the frontal plane, and embedded in paraffin.

### Immunohistochemistry

Paraffin-embedded lung sections (4 μm) were deparaffinized in xylene and rehydrated in ethanol. Heat-induced epitope retrieval (10 min at 125 °C) was performed in citrate buffer (pH 6.0; LSI Medience Corporation, Tokyo, Japan). After blocking with 2% bovine serum albumin in PBS, tissue sections were incubated at 4 °C overnight with anti-COX-2 antibody (1:300; BD Biosciences, San Jose, CA, USA). Tissue sections were then incubated with biotin-labeled anti-mouse IgG antibody (1:300; Agilent Technologies) and labeled with avidin (1:50; Vector Laboratories, Burlingame, CA, USA) for 40 min at between 25 °C and 28 °C. Signals were detected using hydrogen peroxide and 3,3-diaminobenzidine tetrahydrochloride. Tissue sections were counterstained with hematoxylin and dehydrated in xylene. The ratio of positively immuno-stained nuclei to the total count of nuclei present in a field at 400× magnification was determined in 10 different lung areas per mouse.

### Statistical analysis

Data are expressed as the mean ± standard error of the mean (SEM) and were analyzed using GraphPad Prism 6 (GraphPad Software, San Diego, CA, USA). Analysis of variance was performed using a non-parametric Kruskal–Wallis test. When applicable, the Mann–Whitney *U* test was used for comparisons between groups. Differences were considered significant at *p* <  0.05.

## Results

### Gene-expression profiles altered by CSE exposure in SAECs and NHBEs

**Fig. 1:**
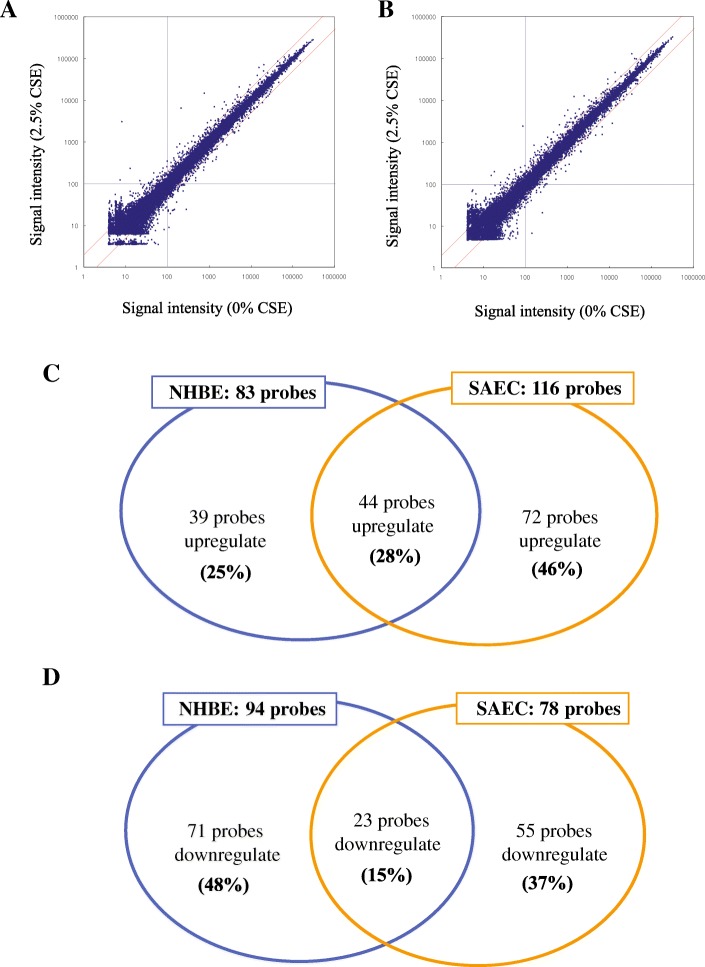
Gene-expression profiles after exposure to CSE in SAECs and NHBEs. Two different batches of SAECs and NHBEs were used. Cells were exposed to 2.5% CSE for 24 h or not (controls). Scatter plot of (**a**) NHBEs and (**b**) SAECs. The vertical axis represents the relative signal intensity in CSE-exposed cells, and the horizontal axis represents the control (without CSE exposure). Both axes show log scales. **c** Venn diagram of probes showing significant upregulation in SAECs and NHBEs after CSE exposure. **d** Venn diagram of probes showing significant downregulation in SAECs and NHBEs after CSE exposure. CSE, cigarette smoke extract; SAECs, small airway epithelial cells; NHBEs, normal human bronchial epithelial cells.

As shown in the scatter plots, exposure to CSE for 24 h altered the expression of several probes in NHBEs and SAECs (Fig. 1a and b). In NHBEs, 83 probes were upregulated (≥ 2-fold) by CSE exposure, while in SAECs 116 probes were upregulated (Fig. 1c). Among upregulated probes, 72 were upregulated only in SAECs (46% of the total upregulated probes), whereas 39 were upregulated only in NHBEs (25%). Ninety-four and 78 probes were downregulated (≤ 2-fold) by CSE exposure in NHBEs and SAECs, respectively, with 71 probes (48% of the total downregulated probes) downregulated in NHBEs alone and 55 (37%) downregulated in SAECs alone (Fig. 1d). Differentially expressed probes are listed in Additional file 1: Tables S1–S6.

To elucidate the biological significance of altered gene-expression profiles in NHBEs and SAECs, we performed GSEA based on two different gene sets chosen using KEGG and GO biological-process analyses. As shown in Table 1, KEGG analysis showed that 10 pathways, including the tumor necrosis factor (TNF)- and nuclear factor (NF)-κB-signaling pathways, were significantly and specifically overrepresented in SAECs subjected to CSE exposure. Additionally, GO analysis revealed that 29 GO terms, mostly related to inflammation, were significantly enriched in SAECs (Table 2). In contrast, no pathways or terms were identified by analysis of enrichment of genes downregulated in either CSE-exposed SAECs or NHBEs. In contrast, two pathways, including the JAK-STAT signaling pathway and arachidonic acid metabolism, and five GO terms related to oxidative stress metabolism, were significantly overrepresented in NHBEs subjected to CSE exposure (Tables 3, 4).

**Table 1:** Overrepresented pathways in SAECs based on KEGG analysis

Pathway	*p*	Genes
Rheumatoid arthritis	< 0.001	*CCL20, MMP1, IL1B, TLR2, FOS, IL8*
TNF-signaling pathway	0.001	*CCL20, PTGS2, IL1B, MAP3K8, FOS, TRAF1*
Toll-like receptor-signaling pathway	0.001	*SPP1, IL1B, MAP3K8, TLR2, FOS, IL8*
Leishmaniasis	0.001	*NCF1, PTGS2, IL1B, TLR2, FOS*
Malaria	0.004	*CSF3, IL1B, TLR2, IL8*
Amoebiasis	0.005	*IL1B, IL1R2, TLR2, IL8, SERPINB2*
NF-κB-signaling pathway	0.020	*PTGS2, IL1B, TRAF1, IL8*
MAPK-signaling pathway	0.026	*NR4A1, PLA2G4A, IL1B, IL1R2, MAP3K8, FOS*
Chagas disease (American trypanosomiasis)	0.032	*IL1B, TLR2, FOS, IL8*

*SAECs* small airway epithelial cells, *KEGG* Kyoto Encyclopedia of Genes and Genomes

**Table 2:** Overrepresented terms in SAECs by GO enrichment analysis

GO term	*p*	Genes
Inflammatory response	< 0.001	*CCL20, SPP1, PTGS2, IL1F9, IL1B, BMP2, TLR2, FOS, ELF3, CYP4F11, IL24, IL8*
Response to vitamin D	< 0.001	*SPP1, PLA2G4A, PTGS2, IL1B*
Response to hypoxia	< 0.001	*SOD2, MUC1, IL1B, CYP1A1, BMP2, ALDH3A1, TLR2*
Positive regulation of fever generation	0.001	*PLA2G4A, PTGS2, IL1B*
Wound healing	0.001	*EREG, CCL20, IL1B, IL24, SERPINB2*
Response to lipopolysaccharide	0.002	*SOD2, PLA2G4A, CCRN4L, PTGS2, CYP1A1, FOS*
Ovulation	0.002	*EREG, PTGS2, IL1B*
Aging	0.003	*NQO1, PLA2G4A, IL1B, CYP1A1, ALDH3A1, FOS*
Cellular response to organic cyclic compound	0.004	*IL1B, CYP1A1, BMP2, CYP1B1*
Positive regulation of apoptotic process	0.004	*ALDH1A3, NR4A1, PLA2G4A, PTGS2, IL1B, BMP2*
Decidualization	0.005	*SPP1, PLA2G4A, PTGS2*
Positive regulation of NF-κB import into nucleus	0.005	*PTGS2, IL1B, TLR2*
Epithelial cell differentiation	0.005	*DHRS9, MUC1, AKR1C1, ELF3*
Arachidonic acid metabolic process	0.007	*CYP4F12, PLA2G4A, CYP1B1*
Positive regulation of vascular endothelial growth factor production	0.007	*PTGS2, IL1B, CYP1B1*
Response to immobilization stress	0.007	*SOD2, CYP1A1, FOS*
Transcription from RNA polymerase II promoter	0.012	*OVOL1, NFYC, ATF3, CCRN4L, PIR, FOSB, FOS, ELF3*
Negative regulation of cell cycle	0.013	*NR4A1, PTGS2, BMP2*
Apoptotic process	0.019	*NCF1, NR4A1, IL1B, MAP3K8, TLR2, TRAF1, MAL, IL24*
Positive regulation of transcription from RNA polymerase II promoter	0.019	*NFYC, NR4A1, ATF3, FOSB, MYO6, CSF3, IL1B, BMP2, TLR2, FOS, ELF3*
Embryo implantation	0.019	*SPP1, PTGS2, IL1B*
Positive regulation of nitric oxide biosynthetic	0.019	*SOD2, PTGS2, IL1B*
Cellular response to lipopolysaccharide	0.023	*CCL20, CSF3, IL24, IL8*
Response to progesterone	0.024	*FOSB, TLR2, FOS*
Skeletal muscle cell differentiation	0.024	*NR4A1, ATF3, FOS*
Response to cAMP	0.027	*FOSB, ALDH3A1, FOS*
Cell-cell signaling	0.033	*EREG, CCL20, IL1F9, IL1B, BMP2*
Positive regulation of gene expression	0.034	*PRDM1, ID1, IL1B, BMP2, TLR2*
Neutrophil chemotaxis	0.040	*CCL20, IL1B, IL8*

*SAECs* small airway epithelial cells, *GO* gene ontology

**Table 3:** Overrepresented pathways in NHBEs based on KEGG analysis

Pathway	*p*	Genes
JAK-STAT signaling pathway	0.029	*CCND1, IL13RA2, LIF, CSF3*
Arachidonic acid metabolism	0.033	*PLA2G3, AKR1C3, GPX2*

*NHBEs* normal human bronchial epithelial cells, *KEGG* Kyoto Encyclopedia of Genes and Genomes

**Table 4:** Overrepresented terms in NHBEs by GO enrichment analysis

GO term	*p*	Genes
Doxorubicin metabolic process	< 0.001	*AKR1C3, AKR1B10, AKR1C1*
Daunorubicin metabolic process	< 0.001	*AKR1C3, AKR1B10, AKR1C1*
Retinoid metabolic process	0.001	*AKR1C3, LRP8, AKR1B10, AKR1C1*
Positive regulation of peptidyl-tyrosine	0.003	*CD4, LIF, LRP8, CSF3*
Response to nutrient	0.038	*NQO1, AKR1C3, ALDH3A1*

*NHBEs* normal human bronchial epithelial cells, *GO* gene ontology

**Fig. 2:**
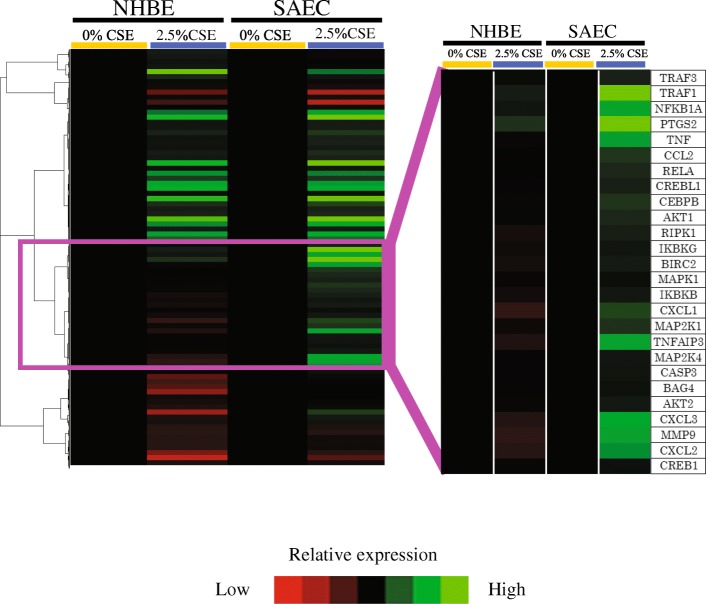
Hierarchical clustering analysis of the TNF-signaling pathway. SAECs and NHBEs were exposed to 2.5% CSE for 24 h or not (controls). Two different batches of SAECs and NHBEs were used. The specific cluster containing genes upregulated by CSE exposure predominantly in SAECs is highlighted (right). High relative expression is indicated in green, and low relative expression is indicated in red. CSE, cigarette smoke extract; SAECs, small airway epithelial cells; NHBEs, normal human bronchial epithelial cells.

Next, we focused on the TNF-signaling pathway and performed hierarchical clustering analysis of the 82 identified genes included in this pathway. As shown in Fig. 2, CSE exposure significantly upregulated genes in a specific cluster in SAECs compared with NHBEs, and the cluster included several inflammation-related genes, including *TNF-receptor-associated factor-1* and *prostaglandin-endoperoxide synthase-2* (*PTGS2*).

### COX-2 expression is altered by CSE exposure in SAECs and NHBEs

**Fig. 3:**
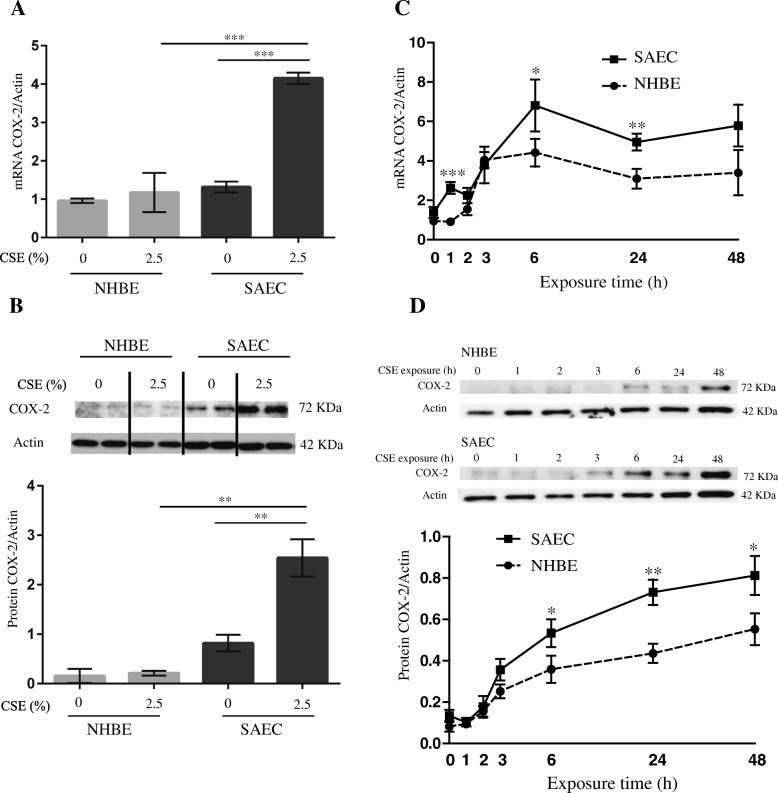
Effects of CSE exposure on COX-2 expression in SAECs and NHBEs. **a** COX-2 mRNA and (**b**) protein expression, with β-actin used as an internal and loading control, respectively. Band size is indicated on the right. Cells were exposed to 2.5% CSE for 24 h or not (controls). **c** Time-course of *COX-2* mRNA expression in SAECs and NHBEs following 2.5% CSE exposure. β-Actin was used as an internal control. **d** Time course of COX-2 protein expression in SAECs and NHBEs following 2.5% CSE exposure. β-Actin was used as a loading control. Band size is indicated on the right. Values are shown as the mean ± SEM. *p <  0.05; **p <  0.01; *** *p* <  0.001 compared with NHBEs in (**c**) and (**d**). Three batches each of SAECs and NHBEs were evaluated on three separate occasions in all experiments. CSE, cigarette smoke extract; SAECs, small airway epithelial cells; NHBEs, normal human bronchial epithelial cells; COX-2, cyclooxygenase-2

Hierarchical clustering analysis revealed that *PTGS2* is highly upregulated after CSE exposure, especially in SAECs. *PTGS2* encodes a key enzyme (COX-2) involved in prostaglandin (PG) biosynthesis. COX-2 is not expressed under normal conditions in most cells; however, elevated levels are observed during inflammation. Furthermore, our previous studies identified a relationship between COX-2 and the abnormal inflammatory response in COPD [16, 17]. Therefore, we evaluated COX-2 mRNA and protein expression following exposure to CSE. After 24 h of exposure, COX-2 mRNA and protein levels were both significantly elevated in SAECs (3.1- and 3.1-fold compared with no CSE treatment, respectively), but not in NHBEs (1.2- and 1.4-fold, respectively) (Fig. 3a and b). To explore the mechanism of COX-2 overproduction in SAECs, we performed time-course analysis of COX-2 expression in response to CSE exposure in NHBEs and SAECs. Our results showed that *COX-2* mRNA levels began to increase 1 h earlier in SAECs than in NHBEs, and that COX-2 production was significantly higher in SAECs than in NHBEs (Fig. 3c). Time-course results for COX-2 protein expression were similar to those obtained for *COX-2* mRNA expression (Fig. 3d).

### COX-2 expression in the lungs of CS-exposed mice

**Fig. 4:**
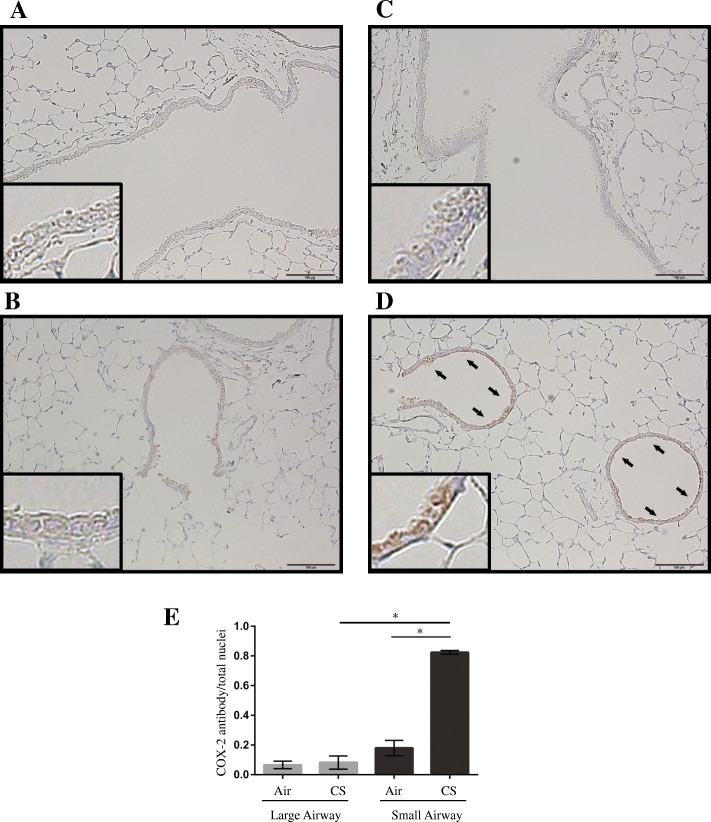
Effects of CS exposure on COX-2 expression in the lungs of C57BL/6 J mice. Mice were exposed to either 3.5% CS or fresh air (control) for 30 min/day for 5 days. Representative immunohistochemistry images of (**a**) the large-airway area in a control lung, (**b**) the small-airway area in a control lung, (**c**) the large-airway area in a CS-exposed lung, and (**d**) the small-airway area in a CS-exposed lung. Brown color indicates COX-2-positive airway epithelial cells (*arrows*). Scale bar, 100 μm. *Insets* are high magnification views of airway epithelial cells. **e** The ratio of COX-2-positive nuclei to the total count of nuclei present in a field at 400× magnification and determined in 10 different areas of the lung per mouse. Values are shown as the mean ± SEM (*n* = 3/group). *p <  0.001. CS, cigarette smoke; COX-2, cyclooxygenase-2

To investigate the initial effect of CS exposure on COX-2 expression in vivo, we performed a short-duration in vivo smoking experiment using C57BL/6 J mice. Immunohistochemical analysis revealed that, after 5 days of exposure to CS, COX-2 expression increased compared with that in air-exposed mice, with more immuno-stained cells observed in small airways than in large airways (Fig. 4a–d). After 5 days of CS exposure, COX-2-positive cells were significantly increased in the small airways of mouse lungs, but not in the large airways (4.6-fold vs. 1.2-fold; Fig. 4e).

## Discussion

This study demonstrated that small and large airways differed in their initial responses to CS exposure. Microarray analysis revealed that small airways showed higher susceptibility to CS compared with large airways and displayed enhanced expression of genes associated with inflammation-related pathways, including TNF-signaling. Among TNF-pathway related genes, *PTGS2*, also known as *COX-2*, showed the greatest difference in expression levels, with higher CSE-induced increases in both mRNA and protein expression in SAECs compared with NHBEs. In vivo short-duration smoking experiments also showed that initial COX-2 expression occurred in lung small-airways. This is the first study clarifying initial genetic and biochemical alterations associated with inflammatory responses to CS exposure in the small and large airways.

CS is the most typical source of exogenous oxidants [18], and oxidants derived from CS can injure airway epithelial cells, leading to persistent inflammation. Oxidative stress and persistent inflammation combine to result in increases in airway free radicals and amplification of the expression of proinflammatory genes that contribute to the release of inflammatory proteins, thereby promoting inflammatory cell infiltration [6]. The present study demonstrated that the expression of proinflammatory genes, and initiation of signaling associated with their related pathways, were more greatly enhanced in SAECs relative to NHBEs following a comparatively short period of CSE exposure. Further, we identified initial induction of *COX-2* expression in the small airway of CS-exposed mice. These results suggest that SAECs represent the initial targets of CS and play an important role in the development of CS-mediated inflammation, which might contribute to CS-related respiratory diseases, such as COPD.

Accumulating evidence suggests that the small airway is the initial locus of inflammation in COPD and plays a critical role in disease development [19]. Based on clinical findings, even patients with mild COPD already show some pathological abnormalities in the small-airway area, including active inflammation and obstruction [9]. Based on the results of multidetector computed-tomography, McDonough et al. [11] reported that small-airway injury precedes the development of centrilobular emphysema, and another study of pathology in smokers who underwent surgical resection showed that the number of neutrophils and mast cells infiltrating the small airway was higher than that in the large airway [20]. In terms of genetic analysis, Mercer et al. [21] performed microarray analyses using CSE-exposed SAECs and showed differential regulation of 425 genes involved in various biological processes, including immune function, signal transduction, apoptosis, the cell cycle, cell proliferation, and antioxidation. The results of the present study were consistent with the findings of Mercer et al. [21], and further demonstrate that CSE exposure affects gene groups related to diverse biological processes in SAECs, including the inflammatory response, wound healing, aging, apoptosis, cell differentiation, and neutrophil chemotaxis (Table 2). Importantly, these findings extend previous results by demonstrating that SAECs exhibit greater alterations in gene regulation and a higher susceptibility to CSE exposure than NHBEs.

A recent study by Yang et al. demonstrated that smoking induced *distal-to-proximal repatterning* of the adult human small airway epithelium, which is a pathologic feature of COPD [22] . The authors showed that the proximal airway epithelium is enriched for molecular features related to oxidative stress, xenobiotic metabolism, and nicotine degradation, suggesting that the proximal airway epithelium can more robustly tolerate oxidants and toxins compared with the small airway epithelium. In the current study, the GSEA in NHBEs indicated that a smaller number of pathways and GO terms were overrepresented following CSE exposure compared to SAECs (Tables 3, 4); however, *GPX-2* [glutathione peroxidase] was one of the enriched genes in the arachidonic acid metabolism pathway, according to KEGG analysis (Table 3). Furthermore, GO enrichment analysis showed that several *AKR* [aldo-keto reductase] family members, which encode enzymes that detoxify oxidative stress-induced carbonyl proteins, were enriched (Table 4). These findings are consistent with those of Yang et al. [22], showing that *GPX-2* is a proximal airway epithelial signature gene. Interestingly, Yang et al. [22] also showed that the expression of proximal signature genes was increased in the small airway epithelium of healthy smokers and COPD patients compared with nonsmokers. They named this phenomenon, which may be relevant to the pathogenesis of small airway disorder in COPD, *distal-to-proximal repatterning*. Here, we investigated the initial response towards CS-exposure to determine whether the small-airway is the initial site of the CS-induced lung damage; however, investigations during longer periods of CSE exposure will likely be fruitful beyond the current study.

Here, we focused on COX-2 as an important factor that initiates an inflammatory response to CSE exposure in the lung. COX-2 is a key mediator of inflammation that catalyzes the transformation of arachidonic acid to thromboxanes and PGs, such as prostaglandin E_2_ (PGE_2_) [23]. COX-2 is also believed to be associated with COPD pathogenesis [24]. A previous animal study showed that treatment with a COX-2 inhibitor attenuated PGE_2_ synthesis and protected against the development of CS-induced pulmonary emphysema [25]. Our previous study, using primary lung fibroblasts from patients with COPD and smokers without COPD (controls), demonstrated that following stimulation with inflammatory cytokines, fibroblasts from COPD subjects produced higher PGE_2_ levels relative to those from control subjects, and that elevated PGE_2_ levels were the result of an increase in COX-2 expression due to alterations in mRNA stability caused by weak induction of microRNA-146a [17]. More recently, Zago et al. demonstrated that expression of RelB, a component of the non-canonical NF-κB pathway, and the aryl hydrocarbon receptor, are both reduced in fibroblasts from COPD patients and regulate COX-2 protein levels by altering mRNA stability [26–29]. These findings suggest that the regulation of COX-2 is critical to controlling abnormal inflammation associated with COPD pathogenesis, and that its targeting could be a promising strategy for COPD therapeutics. Interestingly, a recent study suggested that mesenchymal stem cells attenuated airway inflammation and emphysema in CS-exposed rats through the downregulation of COX-2 and PGE_2_ [30].

This study had several limitations. First, we did not use primary cells from COPD patients to evaluate the direct contribution of the current findings to the disease condition. Second, the SAECs and NHBEs used in the current study were not from matched donors. Instead, we confirmed COX-2 expression in the lungs of CS-exposed mice as an experimental model of COPD. Thirdly, we used different media approved by the supplier for SAECs and NHBEs. Their components are almost identical; however, there is a possibility that they influenced the current findings. Additionally, we did not perform air-liquid interface (ALI) culture to promote differentiation. The phenotypes of each cell type observed under non-differentiating conditions could be affected by contamination with different cell types. Therefore, differentiating SAECs and NHBEs using ALI culture system first and then exposing to CSE will make experimental findings more applicable to human airways. However, our findings were consistent with those from a previous study using ALI culture [22], indicating that commercially available cells cultured using conventional methods can be suitable for these types of experiment. Lastly, we did not define the mechanisms by which inflammation spreads from the initial site to the whole lung, leading to development of pulmonary emphysema. Further investigation of the transition of COX-2 expression in the airways, following various durations of CS exposure, and the interactions between small-airway cells and other cells will be beneficial.

## Conclusions

This study demonstrated that the small airway is more susceptible to CS than the large airway and might represent an initial target site for development of CS-related respiratory diseases, such as COPD. These findings support the results of previous pathological investigations.

## Additional file

Additional file 1: **Table S1**. A list of 44 probes upregulated by CSE exposure in both NHBEs and SAECs. **Table S2.** A list of 72 probes upregulated by CSE exposure only in SAECs. **Table S3.** A list of 39 probes upregulated by CSE exposure only in NHBEs. **Table S4.** A list of 23 probes downregulated by CSE exposure in both NHBEs and SAECs. **Table S5.** A list of 55 probes downregulated by CSE exposure only in SAECs. **Table S6.** A list of 71 probes downregulated by CSE exposure only in NHBEs. (DOCX 3206 kb)

## Abbreviations

ALI: Air-liquid interface
COPD: Chronic obstructive pulmonary disease
COX-2: Cyclooxygenase-2
CS: Cigarette smoke
CSE: Cigarette smoke extract
GO: Gene ontology
GSEA: Gene set enrichment analysis
KEGG: Kyoto Encyclopedia of Genes and Genomes
NF-κB: Nuclear factor-κB
NHBE: Normal human bronchial epithelial cells
PBS: Phosphate-buffered saline
PG: Prostaglandin
PGE_2_: Prostaglandin E_2_
PTGS2: Prostaglandin-endoperoxide synthase 2
SAEC: Small airway epithelial cells
SEM: Standard error of the mean
TNF: Tumor necrosis factor
