# Supplementary material for: Regional heterogeneity in response of airway epithelial cells to cigarette smoke
Source: BMC Pulm Med. 2018 Sep 4;18:148. doi: 10.1186/s12890-018-0715-4 (PMC6122713; doi:10.1186/s12890-018-0715-4)
Supplement: Supplementary file 1 — Table S1. A list of 44 probes upregulated by CSE exposure in both NHBEs and SAECs. Table S2. A list of 72 probes upregulated by CSE exposure only in SAECs. Table S3. A list of 39 probes upregulated by CSE exposure only in NHBEs. Table S4. A list of 23 probes downregulated by CSE exposure in both NHBEs and SAECs. Table S5. A list of 55 probes downregulated by CSE exposure only in SAECs. Table S6. A list of 71 probes downregulated by CSE exposure only in NHBEs. (DOCX 3206 kb) [file 12890_2018_715_MOESM1_ESM.docx]

**Regional heterogeneity in response of airway epithelial cells to cigarette smoke**

Hario Baskoro, Tadashi Sato, Keiko Karasutani, Yohei Suzuki, Aki Mitsui, Naoko Arano, Fariz Nurwidya, Motoyasu Kato, Fumiyuki Takahashi, Yuzo Kodama, Kuniaki Seyama, Kazuhisa Takahashi

**Supplementary Tables**

**Suppl. Table 1. A list of 44 probes upregulated by CSE exposure in both NHBEs and SAECs.**

**
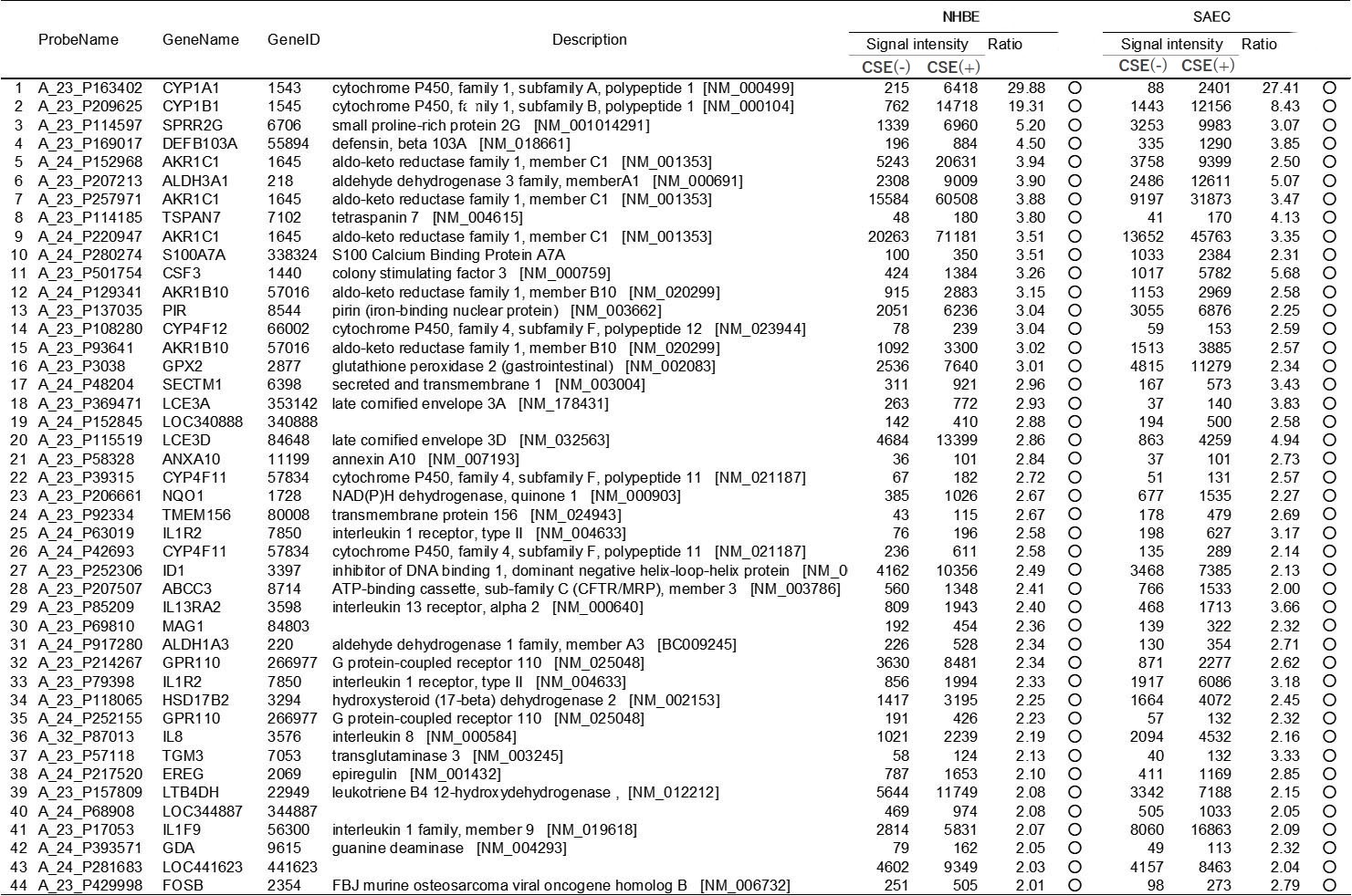
**CSE, cigarette smoke extract; NHBEs, normal human bronchial epithelial cells; SAECs, small airway epithelial cells.


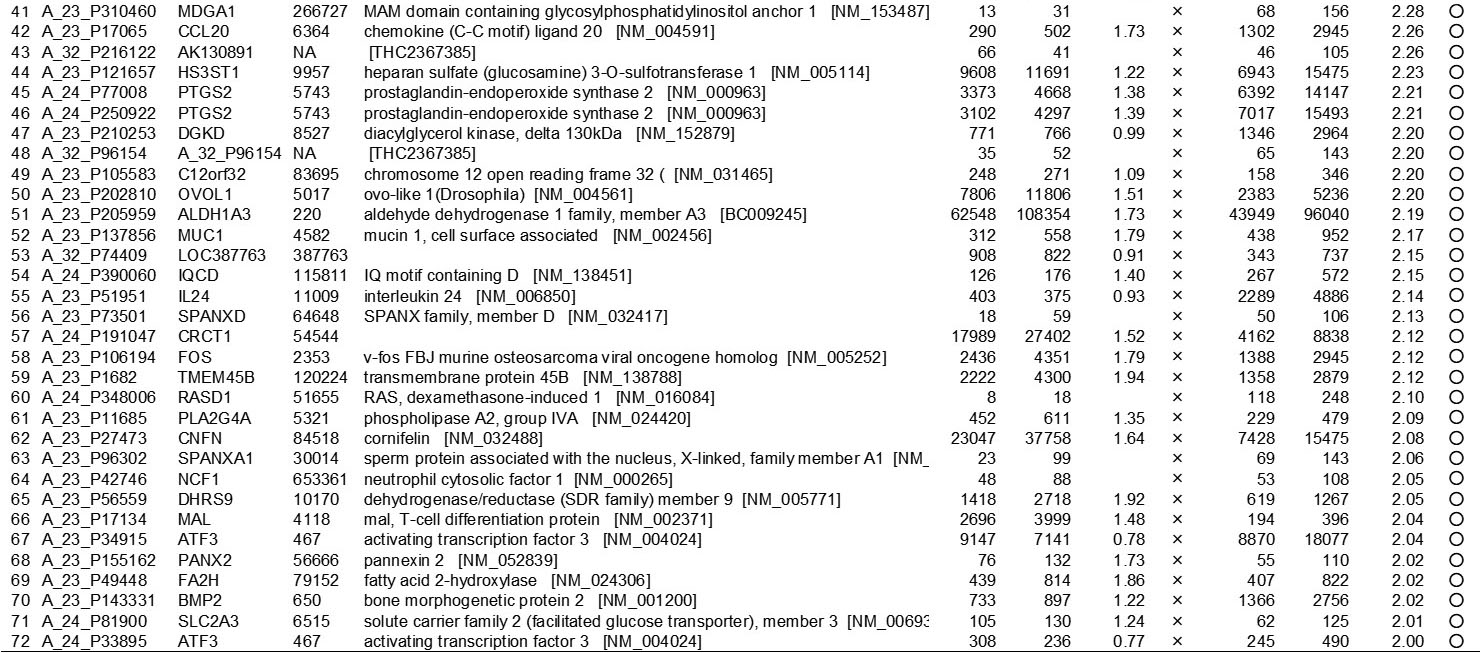

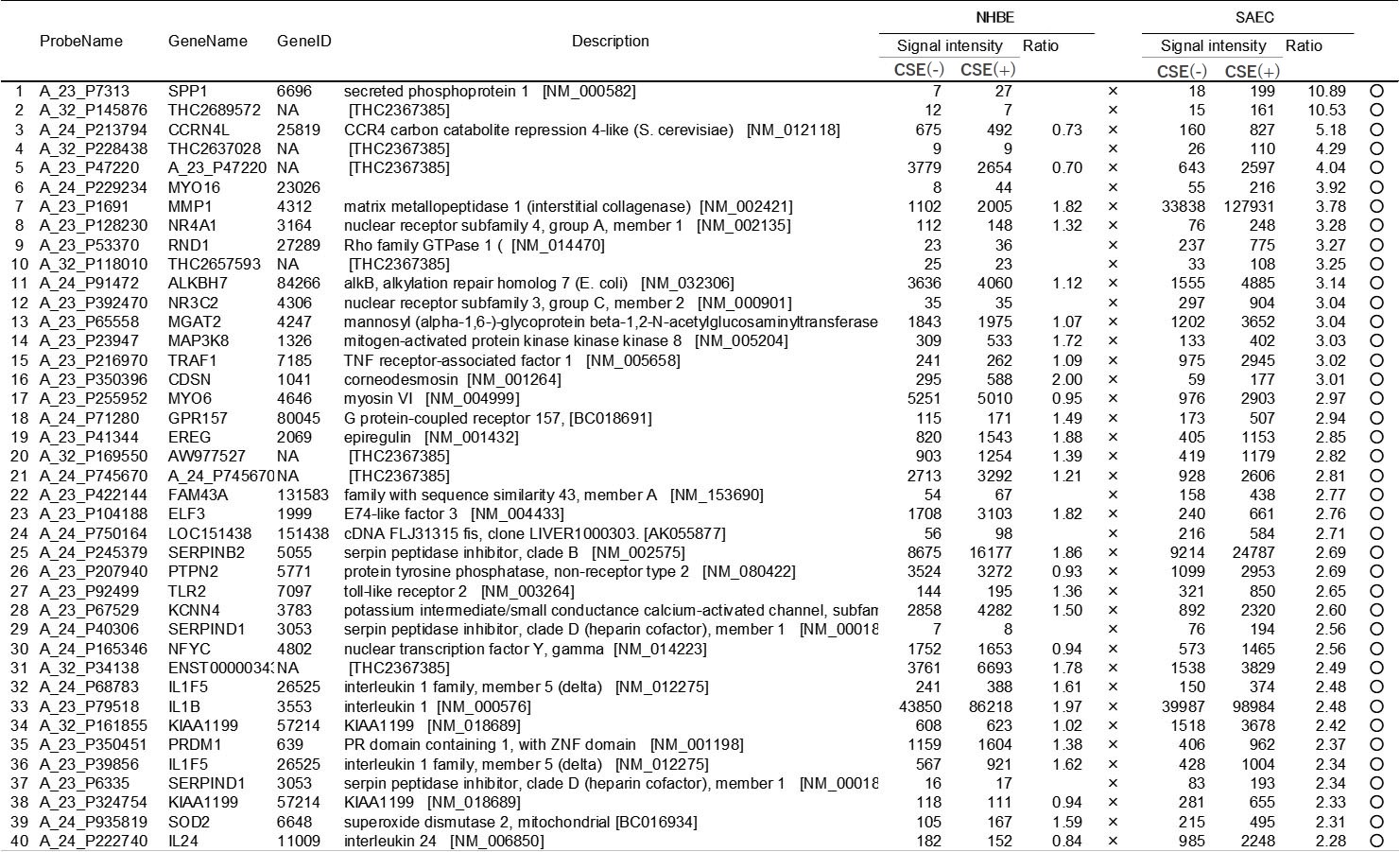
**Suppl. Table 2. A list of 72 probes upregulated by CSE exposure only in SAECs.**

CSE, cigarette smoke extract; NHBEs, normal human bronchial epithelial cells; SAECs, small airway epithelial cells.

**
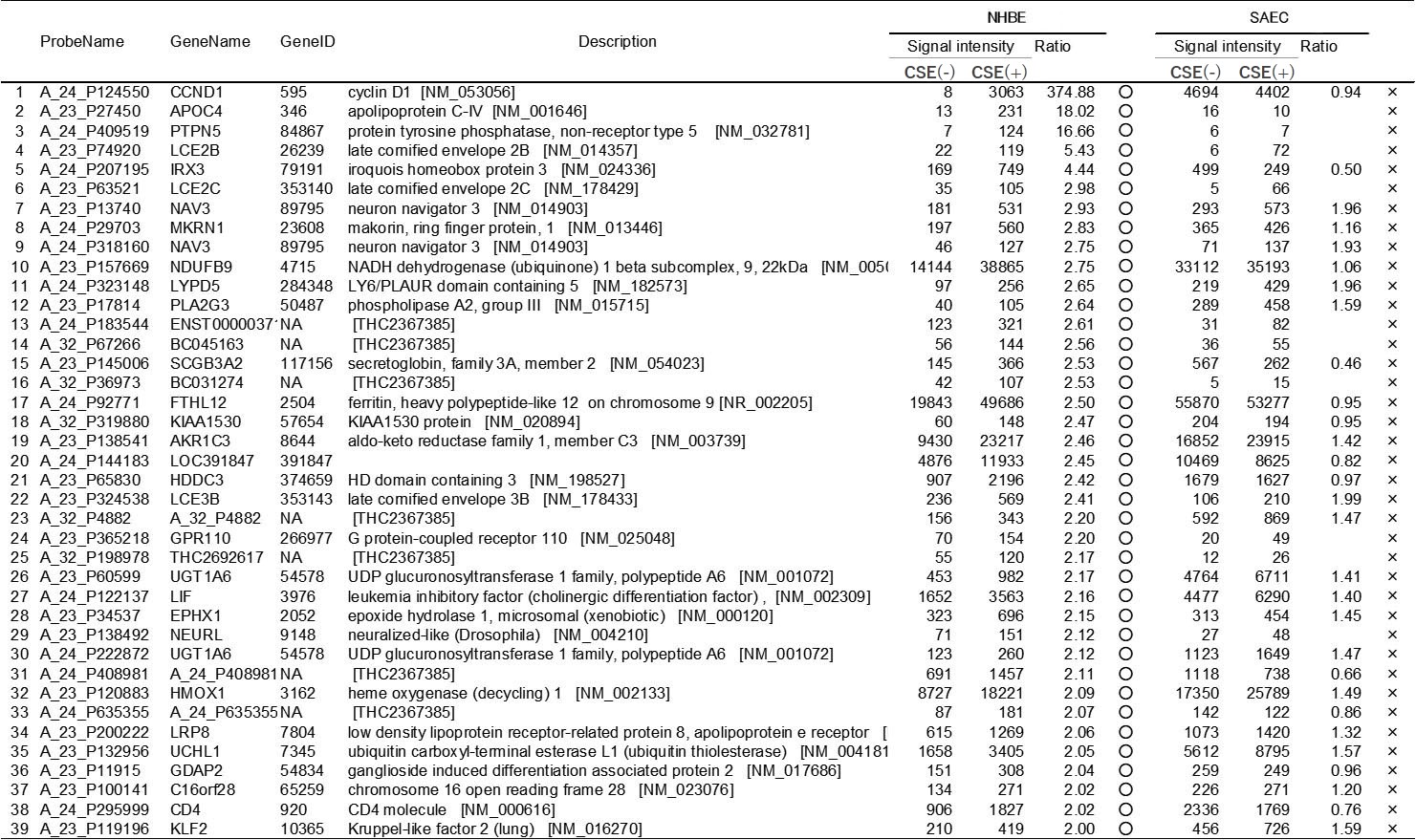
Suppl. Table 3. A list of 39 probes upregulated by CSE exposure only in NHBEs.**

CSE, cigarette smoke extract; NHBEs, normal human bronchial epithelial cells; SAECs, small airway epithelial cells.

**
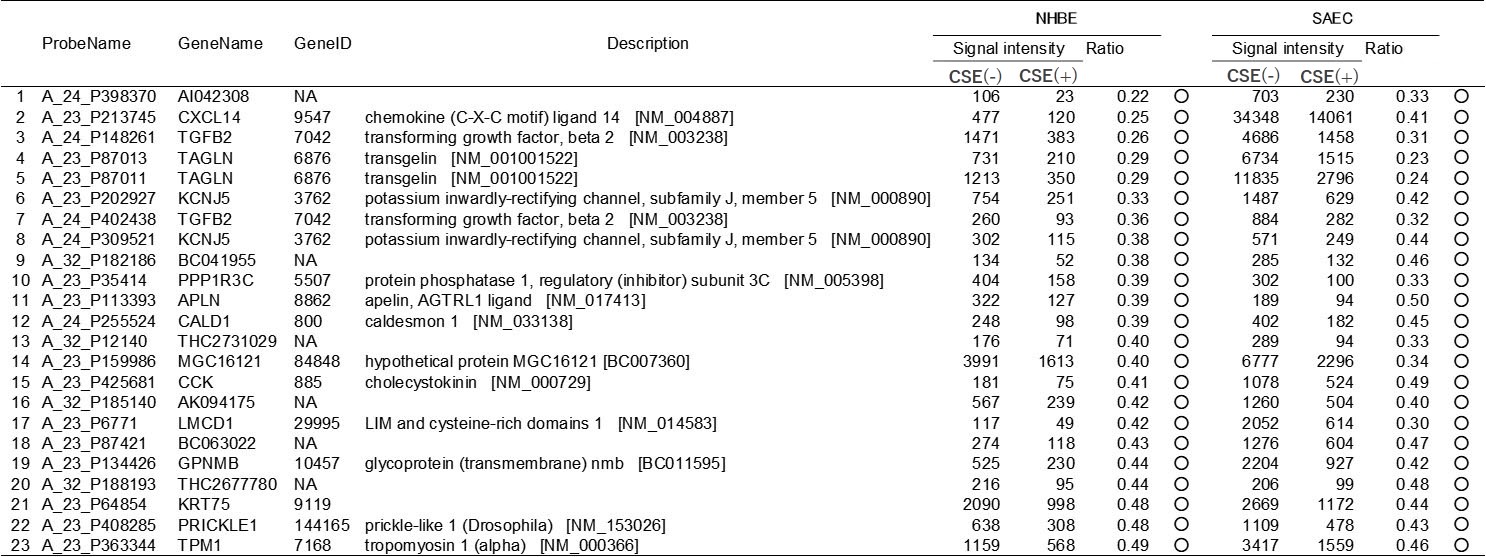
Suppl. Table 4. A list of 23 probes downregulated by CSE exposure in both NHBEs and SAECs.**

CSE, cigarette smoke extract; NHBEs, normal human bronchial epithelial cells; SAECs, small airway epithelial cells.


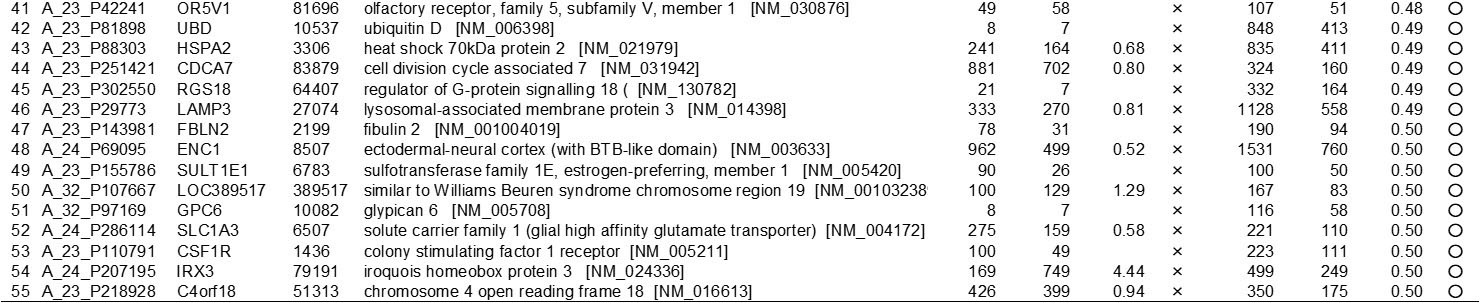

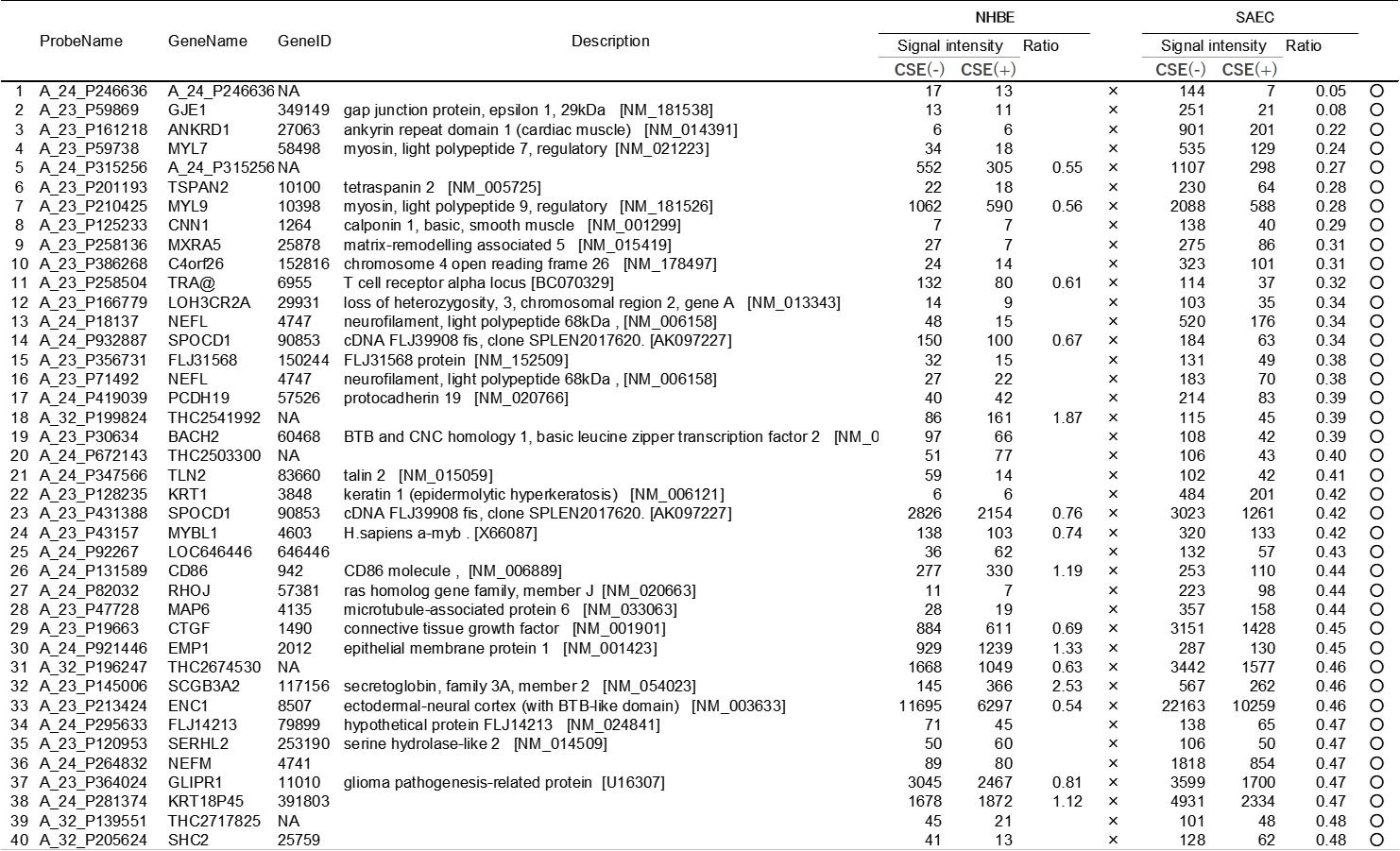
**Suppl. Table 5. A list of 55 probes downregulated by CSE exposure only in SAECs.**

CSE, cigarette smoke extract; NHBEs, normal human bronchial epithelial cells; SAECs, small airway epithelial cells.


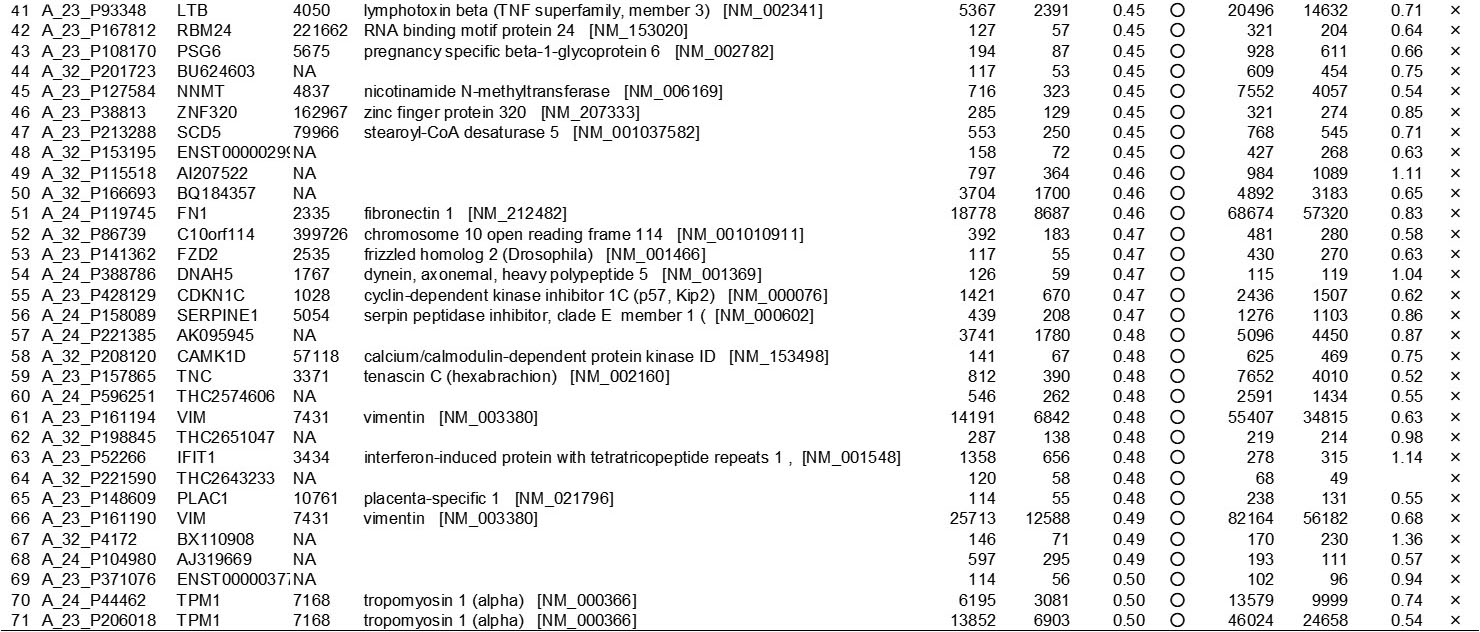
**
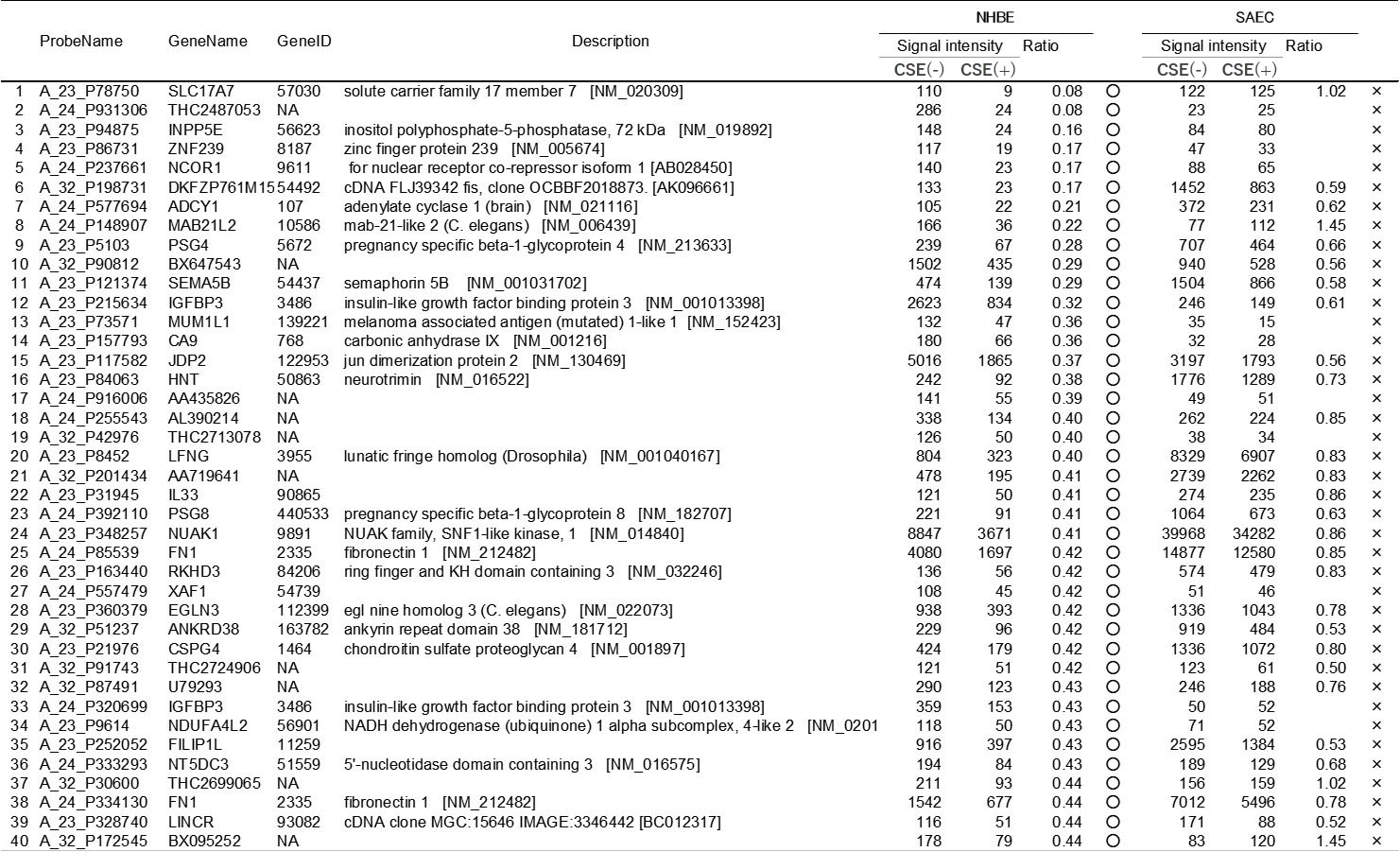
Suppl. Table 6. A list of 71 probes downregulated by CSE exposure only in NHBEs.**

CSE, cigarette smoke extract; NHBEs, normal human bronchial epithelial cells; SAECs, small airway epithelial cells.
